# Evaluating Fellowship Training for Endobronchial Ultrasound-Transbronchial Needle Aspiration (EBUS-TBNA) Using the EBUS-Skills and Task Assessment Tool (STAT) and High-Fidelity Bronchoscopy Simulator

**DOI:** 10.7759/cureus.71810

**Published:** 2024-10-18

**Authors:** Bryan K Dunn, Anagha Malur, Mark Bowling, Kori L Brewer, Jennifer Stahl

**Affiliations:** 1 Pulmonary and Critical Care, East Carolina University, Brody School of Medicine, Greenville, USA; 2 Emergency Medicine, East Carolina University, Brody School of Medicine, Greenville, USA

**Keywords:** ebus-stat (skills and task assessment tool), ebus-tbna (endobronchial ultrasound-transbronchial needle aspiration), high-fidelity simulation, pulmonary fellows, training proficiency

## Abstract

Background: There are two approaches for endobronchial ultrasound (EBUS) training: the traditional apprenticeship approach involving ‘see one, do one, teach one’, and the computer-based simulation approach. In the traditional approach, the trainee learns under direct supervision from an expert preceptor while performing on patients. In the latter approach, trainees use a high-fidelity bronchoscopy simulator, undertake a skills assessment exam (Endobronchial Ultrasound Skills and Task Assessment Tool (EBUS-STAT)), and receive supervised patient-based training from experienced clinicians. The primary objective of this study was to determine whether a single-day EBUS-transbronchial needle aspiration (TBNA) training course, using high-fidelity simulation and the EBUS-STAT tool, increased the confidence of pulmonary fellows for performing EBUS-TBNA procedures.

Methods: Fellowship confidence was measured using a 10-item questionnaire, the EBUS self-assessment tool (EBUS-SAT). This questionnaire was completed before and after EBUS training. The secondary objectives were to determine whether EBUS-STAT varied by year of training and to evaluate the utility of incorporating the EBUS-TBNA training session into fellowship education.

Results: We found that a single day of EBUS-TBNA training using high-fidelity simulation and the EBUS-STAT tool increased the confidence of the first- and third-year pulmonary fellows. The mean improvements in EBUS-SAT questionnaire scores pre- and post-EBUS training session were 13.3 points (p = 0.002) for the first-year fellows and 8.5 points (p = 0.0001) for the third-year fellows. Regarding the secondary objectives, we found no significant difference between EBUS-STAT scores of the first- and third-year pulmonary fellows (p = 0.71).

Conclusions: Combining high-fidelity bronchoscopy simulator training with the EBUS-STAT assessment tool was found to benefit and improve training in EBUS-TBNA and should be incorporated into fellowship training programs.

## Introduction

Bronchoscopy has helped clinicians improve patient care since the late 1800s. Dr. Gustav Killian, a German laryngologist and pioneer in the field of respiratory medicine, is considered to be the father of bronchoscopy; in 1887, he successfully conducted the first bronchoscopic procedure to inspect the trachea and primary bronchi, employing a rigid laryngoscope. This procedure not only marked a significant advance in medical diagnostics but also enabled him to perform the first recorded extraction of a foreign body from a patient’s main bronchus [1]. Chevalier Jackson, an otolaryngologist in Philadelphia, is referred to as the father of American bronchoesophagology and was a pioneer in the development of the rigid bronchoscope. In 1904, Dr. Jackson removed over 2,000 objects from his patients’ airways [2,3]. Shigeto Ikeda, a Japanese physician, is considered the father of fiberoptic bronchoscopy and developed the first flexible bronchoscope in 1966. Dr. Ikeda’s motto was “There is more hope with a bronchoscope” and his credo was to “Never give up” [2,4].

Endobronchial ultrasound (EBUS) was first developed in the 2000s, has been widely used for the diagnosis and staging of lung cancer with mediastinal and hilar lymphadenopathy, and is a safe and minimally invasive alternative to the more traditional surgical procedure of mediastinoscopy [5]. EBUS procedures utilize two different probe types: a convex linear probe (CP or CP-EBUS) and a radial probe (RP or r-EBUS). CP-EBUS is a safe and minimally invasive alternative procedure to invasive mediastinoscopy and is currently the most frequently used technique for the diagnosis and sampling of mediastinal and hilar lymphadenopathy [5,6]. When discussing CP-EBUS, we will use the standard accepted terminology, endobronchial ultrasound-transbronchial needle aspiration (EBUS-TBNA). Pulmonologists and thoracic surgeons perform thousands of EBUS-TBNA procedures annually, and the approach has transformed the fields of bronchoscopy and lung cancer.

Lung cancer is the third most prevalent cancer and the leading cause of cancer-related deaths in the United States [7]. Every year 197,000 patients are diagnosed with lung cancer and 136,000 die from this disease, which is equivalent to a commercial plane crashing every day, underlining that adequate training in performing EBUS-TBNA procedures is essential [7]. The Eighth Edition of the TNM (Tumor, Node, Metastasis) system for non-small cell lung cancer utilizes endobronchial ultrasound (EBUS) for diagnosis and staging, particularly for node classification (N) [8]. The TNM system stages (N0, N1, N2, N3) are important for predicting prognosis and guiding treatment strategies [8,9]. According to the American College of Chest Physicians guidelines, EBUS-TBNA is the preferred initial staging method due to its safety, high sensitivity (89%), and a median negative predictive value of 91% [8-10]. In this study, we assessed a standard curriculum based on a previously validated EBUS-TBNA training tool, the “Endobronchial Ultrasound Skills and Task Assessment Tool” (EBUS-STAT), along with high-fidelity bronchoscopy simulation, for EBUS-TBNA training of the first- and third-year pulmonary fellows [11-14].

## Materials and methods

The current standard of care for staging non-small cell lung cancer (NSCLC) involves the use of EBUS-TBNA, with classification into stages I-IV based on the Eighth Edition of the TNM (Tumor, Node, Metastasis) system [8]. In this observational study, we analyzed findings from a four-year experience in EBUS-TBNA training among pulmonary fellows in an Accreditation Council for Graduate Medical Education (ACGME)-accredited pulmonary fellowship. The training model included a three-hour session utilizing the EBUS-STAT assessment tool, which comprises a 10-item exam and the EBUS self-assessment tool (EBUS-SAT) questionnaire, along with a high-fidelity bronchoscopy simulation (Table 1). Our training model included a one-hour PowerPoint lecture on EBUS-TBNA followed by hands-on practice using a high-fidelity bronchoscopy simulator using the EBUS-STAT. Trainees completed a pre- and post-training EBUS-SAT questionnaire which evaluates their confidence in performing these procedures. This course format allowed fellows to receive real-time feedback from experienced faculty on their performance during simulated EBUS-TBNA cases, ensuring a thorough evaluation of their competencies.

**Table 1:** Sequence of EBUS-TBNA fellowship training course. EBUS-TBNA: endobronchial ultrasound-transbronchial needle aspiration, EBUS-SAT: EBUS self-assessment tool.

Completed prior to the course	Completed same day of course	Completed after course
Pre-EBUS-SAT questionnaire	EBUS-TBNA 1 h lecture	Three EBUS-TBNA cases on high-fidelity simulator
EBUS-STAT exam items 8, 9, and 10	EBUS-STAT exam items 1-7, completed using a high-fidelity bronchoscopy simulator	Post-EBUS-SAT questionnaire after performing 3 EBUS-TBNA patient cases

The first-year pulmonary fellows and the more experienced third-year pulmonary fellows were included. The first-year fellows took the course at the end of their first year of training, after they had learned basic bronchoscopy skills and performing at least 10 EBUS-TBNA procedures. The third-year pulmonary fellows underwent the course after two years of bronchoscopy training and experience, after performing at least 20 EBUS-TBNA procedures. This study included 18 pulmonary fellows, comprising 10 first-year fellows and eight third-year fellows. All trainees took the EBUS-TBNA course as part of their three-year pulmonary fellowship training program, from 2020 to 2024. The single-day training session incorporated a one-hour tutorial lecture, followed by high-fidelity bronchoscopy simulation and application in simulated cases (Table 1). The proficiency of the trainee was assessed using the EBUS-STAT 10-point assessment tool. The confidence level of fellows was evaluated by the completion of the EBUS-SAT questionnaire, both pre- and post-training, which was a modification from its original format of a single pre-survey application [13]. The primary objective of this study was to evaluate the confidence levels of fellows pre- and post-EBUS-TBNA training (p < 0.05 was considered significant). Secondary objectives were to determine if the EBUS-STAT 10-item assessment score varied according to year of training and to evaluate the utility of incorporating the EBUS-STAT exam in fellowship training as a teaching tool (p < 0.05 was considered significant) [11,13,15-21].

**Figure 1:**
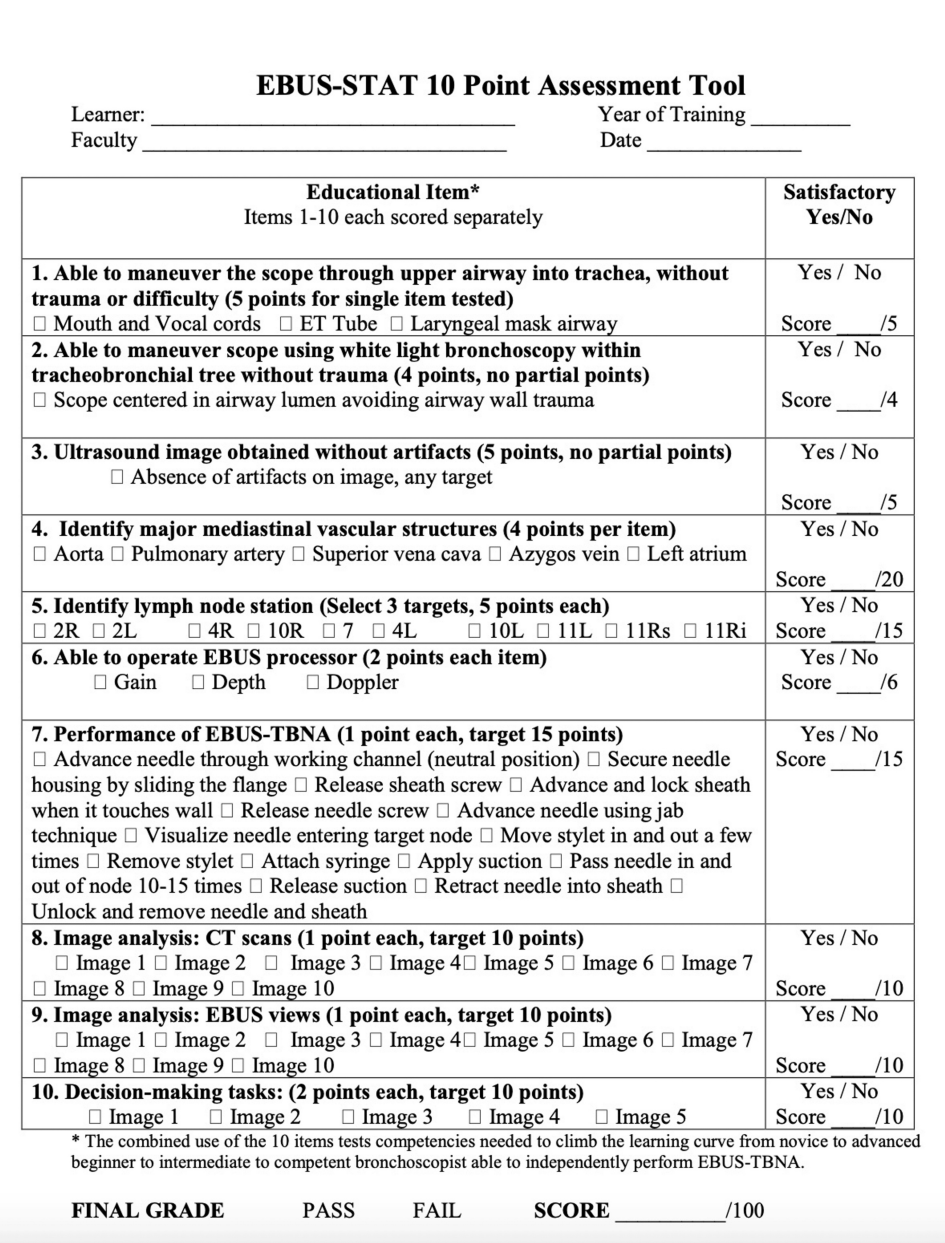
EBUS-STAT (Endobronchial Ultrasound Skills and Task Assessment Tool), 10-Point Assessment Tool. EBUS-TBNA: endobronchial ultrasound-transbronchial needle aspiration, ET: endotracheal. Obtained permission for publication and adapted from https://www.bronchoscopy.org/downloads/BEP%20EBUS%20assessment%20tools.pdf [13].

**Figure 2:**
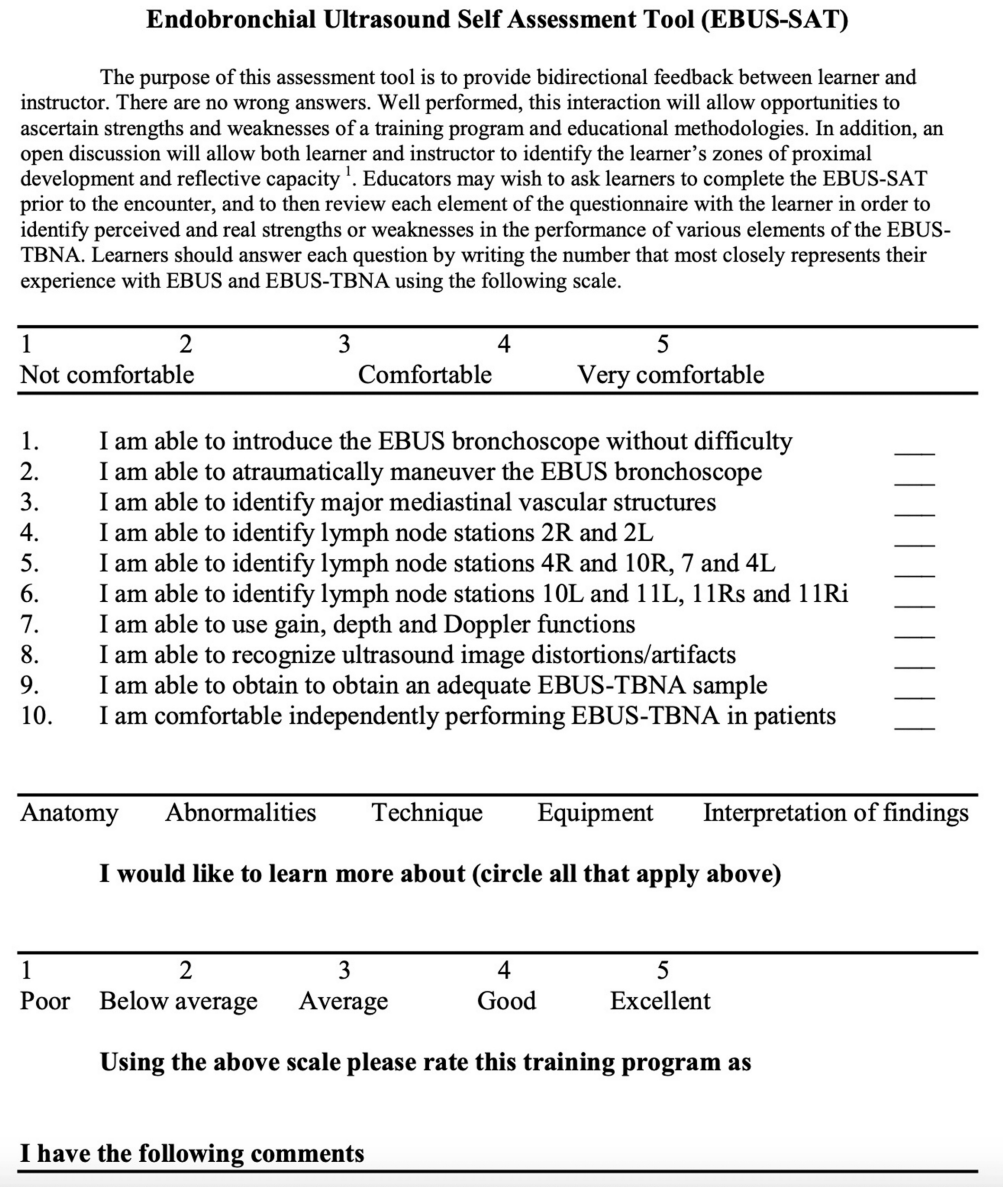
The EBUS-SAT (Endobronchial Ultrasound Self-Assessment Tool) 10 item-questionnaire completed pre- and post-EBUS-TBNA course. EBUS-TBNA: endobronchial ultrasound-transbronchial needle aspiration. Obtained permission for publication and adapted from https://www.bronchoscopy.org/downloads/BEP%20EBUS%20assessment%20tools.pdf [13].

The EBUS-STAT is a an open-access assessment tool used to evaluate learners undergoing EBUS-TBNA training that is published online (http://www.bronchoscopy.org) (Henri Colt, MD), and consists of two parts: the EBUS-SAT self-assessment questionnaire and a 10-item assessment exam (Figures 1, 2) [13]. The EBUS-SAT questionnaire contains 10 questions assessed with scores of 1 through 5 (ranging from not comfortable, to comfortable, to very comfortable) and inquires if the trainees want to learn more about anatomy, abnormalities, technique, equipment, and interpretation of findings. Total scores range from 10 to 50 (Figure 2) [13].

In our study, we modified the original format of the EBUS-SAT questionnaire, which was previously only administered before the EBUS-STAT exam. We administered the EBUS-SAT questionnaire both before the EBUS-TBNA training course and after the completion of at least three EBUS-TBNA patient cases. To our knowledge, this is the first publication to utilize this approach [13].

The EBUS-TBNA fellow training course was administered by an experienced faculty physician who has performed over 1,000 EBUS-TBNA cases and regularly trains both fellows and faculty using the EBUS-STAT system and a high-fidelity simulator. The EBUS-TBNA training session begins with the fellows completing the pre-EBUS-SAT self-assessment questionnaire and EBUS-STAT items 8-10. Following the questionnaire, the training session proceeded with a one-hour tutorial session that introduced EBUS. After the lecture, EBUS-STAT 10-point assessment items 1-7 were completed using the high-fidelity simulator, followed by three simulation cases, then the post-EBUS-SAT questionnaire (Table 2).

**Table 2:** EBUS simulation cases on Endo-Suite BRONCH Mentor high-fidelity simulator (A, B, C). Images taken directly off the screen of the BRONCH Mentor simulator machine. EBUS: endobronchial ultrasound. Reproduced with permission from Surgical Science Sweden AB, Sweden. Permission granted via email by Hans Uddenberg, Director of Product Marketing, Surgical Science (February 2, 2024).

A: Case one: 73-year-old male, with cough, weight loss and hoarseness, former smoker (20 cigarettes per day for 35 years), quit smoking 11 years prior. CT chest showed left lower lobe mass 3.6 x 2.4 cm and lymphadenopathy at stations 4L (left lower paratracheal), station 7 (subcarinal), left hilar. A prior bronchoscopy did not get a diagnosis. Blood pressure 130/92 mmHg, heart rate 98 bpm, respiratory rate 16 breaths/minute.
B. Case two: 72-year-old male diagnosed with squamous cell carcinoma of right upper lobe. CT scan chest showed lymphadenopathy at station 2R (right upper paratracheal), 4R (right lower paratracheal), station 7 (subcarinal) and 10R (right hilar). Blood pressure 136/100 mmHg, heart rate 94 bpm, respiratory rate 12 breaths/minute.
C. Case three: 35-year-old female with cough, fatigue and conjunctivitis, and otherwise healthy. CT scan of chest showed bilateral enlarged mediastinal, hilar and interlobar lymph nodes. Blood pressure 128/80 mmHg, heart rate 92 bpm, respiratory rate 12 breaths/minute.

The EBUS-STAT exam itself includes a 10-item checklist, with items 1-2 testing basic bronchoscopy skills, including whether the trainee can maneuver the scope through the upper airway into the trachea and tracheobronchial tree without causing trauma. Items 3-7 test skill in using ultrasound images without artifacts, identifying major mediastinal vascular lymph nodes (station 2R, 2L, 4R, 4L, 7, 10R, 10 L, 11 Ri, 11Rs, and 11L), operating the processor, and performing the EBUS procedure (Figure 1).

**Figure 3:**
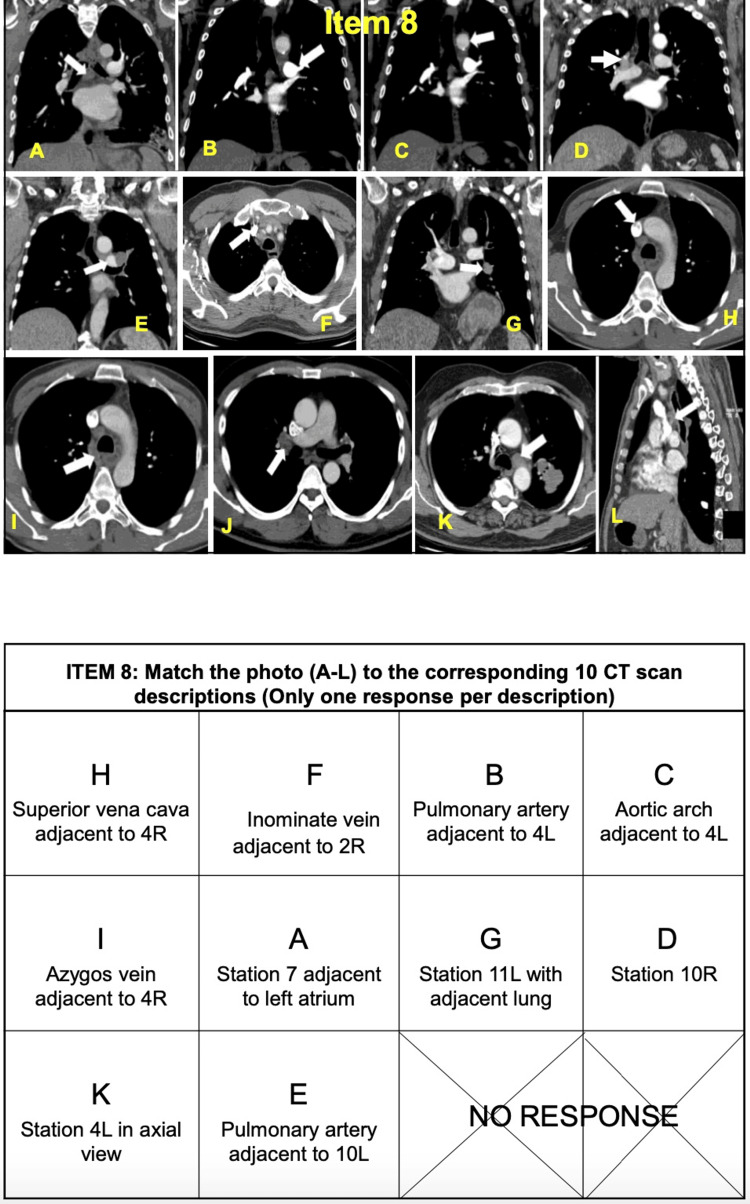
EBUS-STAT items 8 (CT scan interpretation). EBUS-STAT: Endobronchial Ultrasound Skills and Task Assessment Tool. Obtained permission for publication and adapted from https://www.bronchoscopy.org/downloads/BEP%20EBUS%20assessment%20tools.pdf [13].

**Figure 4:**
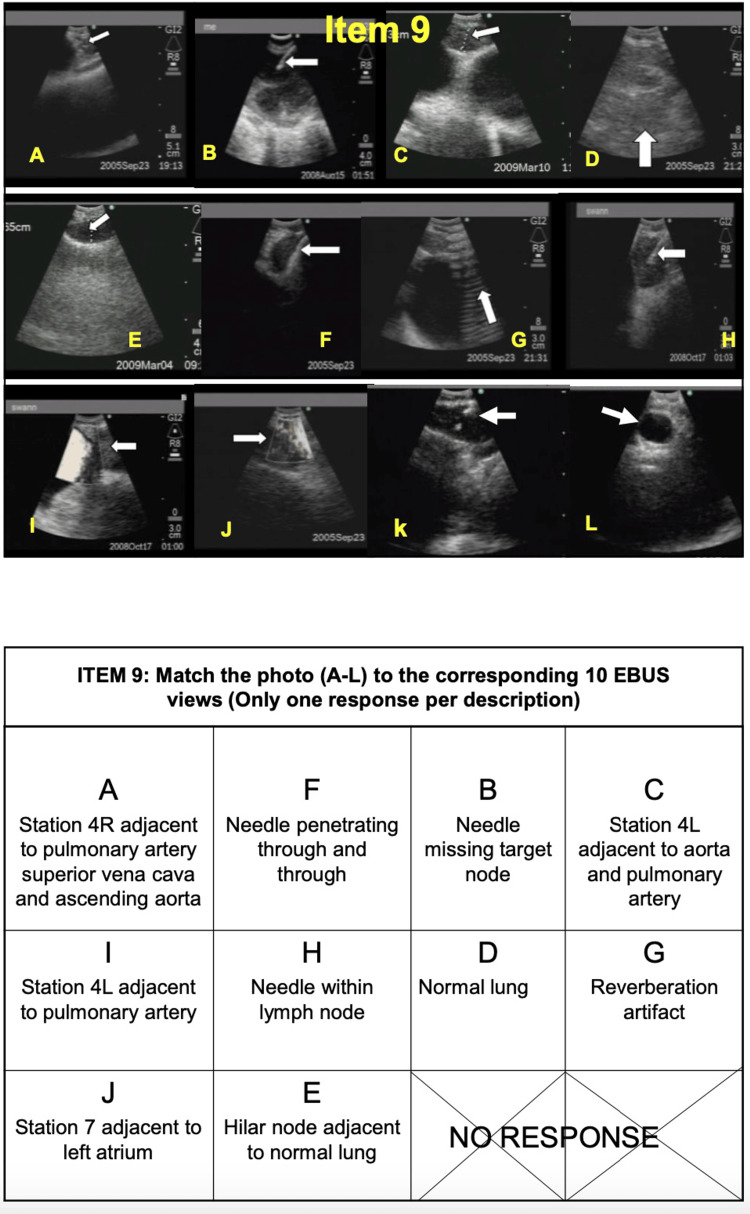
EBUS-STAT item 9 (EBUS image interpretation). EBUS-STAT: Endobronchial Ultrasound Skills and Task Assessment Tool. Obtained permission for publication and adapted from https://www.bronchoscopy.org/downloads/BEP%20EBUS%20assessment%20tools.pdf [13].

**Figure 5:**
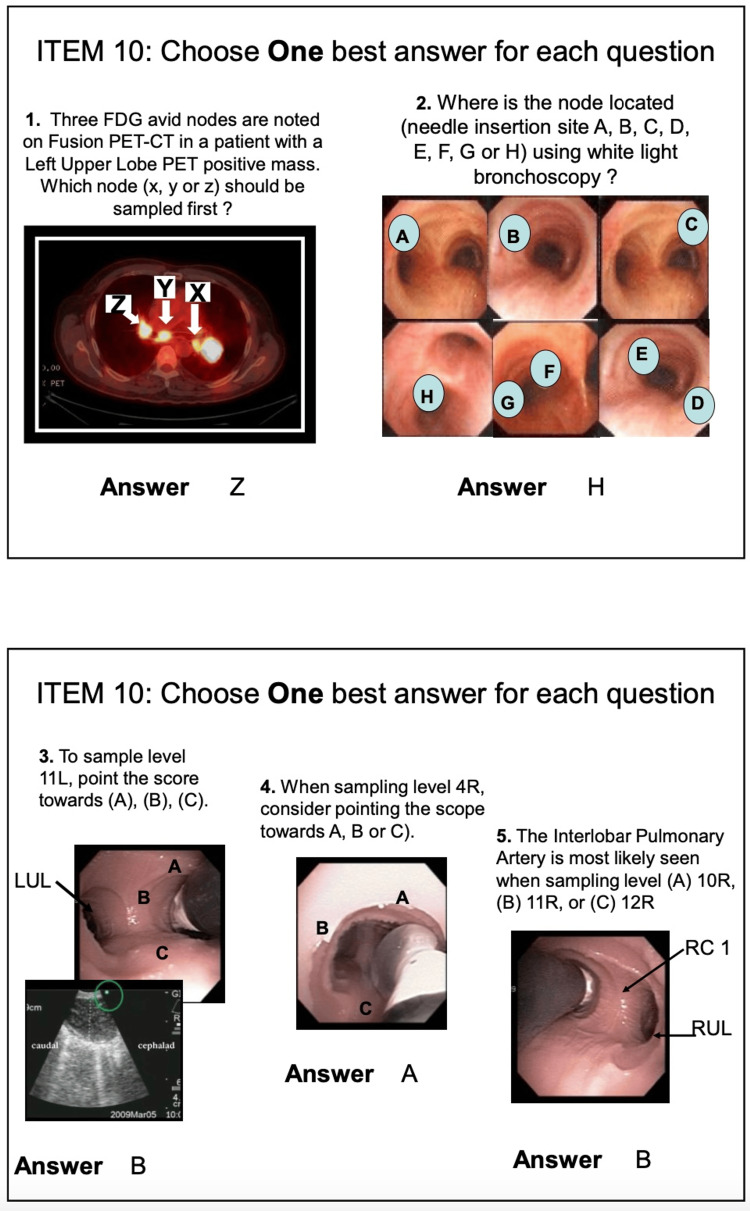
EBUS-STAT item 10 (decision-making skills). EBUS-STAT: Endobronchial Ultrasound Skills and Task Assessment Tool, FDG: fluorodeoxyglucose, PET: positron emission tomography, LUL: left upper lobe. Obtained permission for publication and adapted from https://www.bronchoscopy.org/downloads/BEP%20EBUS%20assessment%20tools.pdf [13].

Items 8, 9, and 10 include questions reviewing CT chest images, EBUS images, and decision-making skills; these last three items are to be completed prior to the day of training (Figures 3-5) [13].

CT chest and EBUS-TBNA images each have 12 photos and 10 matching answers (Figures 3, 4) [13]. The decision-making section has five questions, covering topics including positron emission tomography (PET) scan interpretation and bronchoscopy position-based endobronchial anatomical lymph node locations (Figure 5) [13]. EBUS-STAT exam scores were classified as follows: novice < 60, advanced beginner 60-79, intermediate 80-99, and competent 100 (Figure 2); a score of 100 is recommended before trainees perform EBUS in clinical practice. After completing the EBUS-STAT exam, trainees worked through three high-fidelity simulator EBUS-TBNA cases. The three bronchoscopy simulation cases covered EBUS at varying lymph node stations (Table 2). After completion of the case-based simulations and performing at least three EBUS-TBNA patient cases, a post-EBUS-SAT questionnaire was repeated by the fellows, to evaluate any changes in confidence (Figure 2).

## Results

Prism 7 software (GraphPad, Inc, San Diego, CA) was used to calculate primary and secondary endpoints. The primary endpoint was determining the significance of differences between EBUS-SAT pre- and post-questionnaire scores for the first-year and third-year pulmonary fellows after undergoing a single-day training course using the EBUS-STAT exam, high-fidelity bronchoscopy simulator, accompanying EBUS-TBNA simulator cases, and at least three patient cases and was assessed using a paired two-tailed t test (significance set at p < 0.05). Data are presented as means with standard deviation (SD), graphically represented as scatter dot plots.

**Figure 6:**
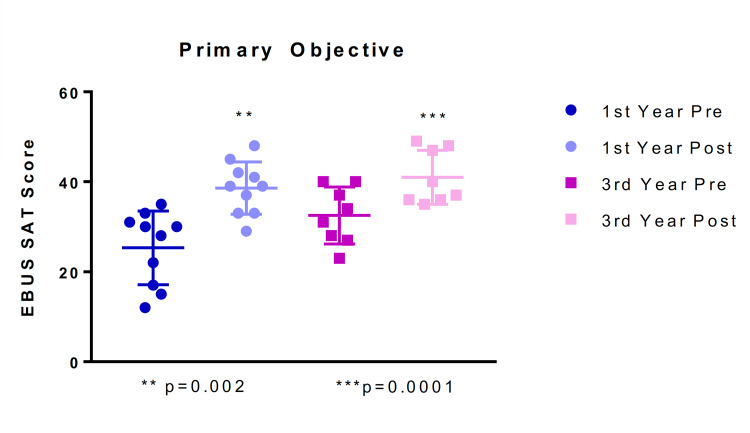
The primary objective was to measure the confidence of the first- and third-year pulmonary fellows using pre- and post-EBUS-SAT questionnaire scores (10-50). EBUS-SAT: endobronchial ultrasound self-assessment tool. Data are graphically represented as means with standard deviation (SD) in a dot plot.

There was a significant improvement in post-EBUS-SAT scores for both the first- and third-year fellows after the EBUS course, lecture, exam, and cases. For the first-year fellow group (N = 10), the mean EBUS-SAT score improvement was 13.3 points (range: 1-31) and the difference was significant (p = 0.0021). The third-year pulmonary fellows (N = 8) had a mean EBUS-SAT score improvement of 8.5 points (range: 5-14), which was also significant (p = 0.0001) (Table 3; Figure 6).

**Table 3:** Primary objectives: pre- and post-EBUS-SAT questionnaire score (10-50) for pulmonary fellows. EBUS-SAT: endobronchial ultrasound self-assessment tool. N = 18 (10 first-year and 8 third-year pulmonary fellows).

Fellow #	Fellow year (first or third)	Pre-EBUS-SAT (10-50)	Post-EBUS-SAT (10-50)	Change in EBUS-SAT	p-value
1	1st	30	39	9	
2	1st	33	45	12	
3	1st	28	29	1	
4	1st	12	37	25	
5	1st	15	33	18	
6	1st	17	48	31	
7	1st	22	42	20	
8	1st	35	41	6	
9	1st	31	39	8	
10	1st	30	33	3	
				Mean 13.3	p = 0.002
11	3rd	40	48	8	
12	3rd	23	37	14	
13	3rd	34	40	6	
14	3rd	31	36	5	
15	3rd	28	35	7	
16	3rd	37	49	12	
17	3rd	27	36	9	
18	3rd	40	47	7	
				Mean 8.5	p = 0.0001

**Figure 7:**
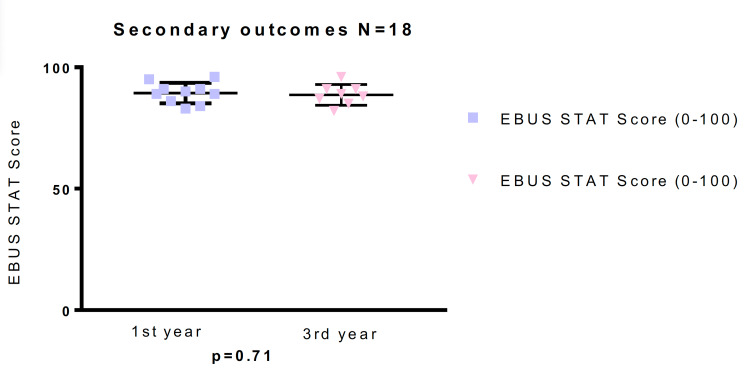
Secondary objectives, comparing EBUS-STAT scores (0-100) between the first-year and third-year pulmonary fellows, p = 0.71. EBUS-STAT: Endobronchial Ultrasound Skills and Task Assessment Tool. Data are graphically represented in a dot plot.

Secondary endpoints included comparison of EBUS-STAT scores between the first- and third-year pulmonary fellows. The first-year fellows had a mean EBUS-STAT score of 89.4 (range: 83-96) while that of the third-year fellows was 88.63 (range: 82-96); there was no significant difference in EBUS-STAT scores between the first- and third-year pulmonary fellows (p = 0.71) (Table 4; Figure 7).

**Table 4:** Secondary objectives: EBUS-STAT score (0-100) according to pulmonary fellow year of training. EBUS-STAT: Endobronchial Ultrasound Skills and Task Assessment Tool. (p = 0.71).

Fellow #	Fellow Year (first or third)	EBUS-STAT score (0-100)
1	1st	86
2	1st	90
3	1st	89
4	1st	89
5	1st	84
6	1st	83
7	1st	95
8	1st	96
9	1st	91
10	1st	91
Mean		89.4
11	3rd	88
12	3rd	91
13	3rd	89
14	3rd	85
15	3rd	82
16	3rd	87
17	3rd	91
18	3rd	96
Mean		88.63
N = 18		p = 0.71

## Discussion

The two available endobronchial ultrasound probes, the RP-EBUS and CP-EBUS, have been in use since 2002 [19]. RP-EBUS is used to help locate pulmonary nodules, but other tools and modalities are required to obtain a tissue biopsy, for example, biopsy forceps, needles, fluoroscopy, and electronic navigation bronchoscopy [16]. In comparison, CP-EBUS is used to locate intrathoracic lymph nodes (mediastinal and hilar lymphadenopathy), followed by real-time lymph node sampling using a convex linear ultrasound probe and TBNA for lung cancer diagnosis and staging (Figures 3, 4).

For EBUS training, prior guidelines from the American College of Chest Physicians recommended that trainees complete 50 supervised EBUS procedures, while the American Thoracic Society has recommended 40 supervised EBUS procedures, prior to independent practice [18]. These guidelines were for RP-EBUS, not CP-EBUS, and were based on expert opinion [17,19,20,21]. In 2016, the CHEST guidelines and Expert Review Panel (Wahidi et al.) recommended using low- or high-fidelity simulation for EBUS-TBNA training, in conjunction with a validated EBUS skills assessment tool (Grade 2C), followed by evaluation and real-time training by expert users, and recommends moving away from volume-based competency assessment for trainees [16,17].

The EBUS-STAT has been reviewed and validated in multiple prior publications [12,13,15,17]. These recommendations were also supported by our prior publication in Clinical Pulmonary Medicine (2019) [15]; we found that a standard curriculum of metrics for EBUS training should include use of well-validated assessment tools, such as EBUS-STAT, with high-fidelity bronchoscopy simulators, followed by training by an experienced clinician. We also found that setting a minimum number of procedures to obtain competency may be insufficient, which is consistent with the 2016 CHEST guidelines authored by Wahidi et al. [16]. Sehgal et al. published a systematic review of EBUS-TNBA training and proficiency in 2017 and found that the number of EBUS-TBNA procedures required to become efficient ranged from 10 to 100 (mean, 37-44 procedures) [10]. In addition, the study found that EBUS assessment tools with high-fidelity bronchoscopy simulators were useful for EBUS training and recommended that training centers implement assessment tools and simulation training into their curriculum [11].

Similar to our investigation, another study reported by Durairajan et al. in 2022 assessed the impact of a multimodal simulation-based curriculum on EBUS-TBNA skills [20]. The authors reviewed 11 first-year pulmonary fellows and used a three-part assessment tool to measure EBUS-related knowledge, procedural skills, and overall self-confidence, using the EBUS-STAT exam and a bronchoscopy simulator, and found that this three-part approach improved confidence, knowledge, and overall procedural skills. In 2017, Scarlata et al. assessed the validity of the EBUS-STAT for evaluating 15 experienced bronchoscopists, with no or limited EBUS-TBNA experience [12]. They reported that the EBUS-STAT, administered before and after a seven-hour virtual simulation training course, could predict trainee improvement and found that learners with a pre-score > 79 remained unchanged after the course; thus, learners with an initial pre-score < 79 likely represent clinicians who would benefit from a simulation training course [12,13]. In 2012, Davoudi et al. assessed the validity of the EBUS-STAT based on level of experience [11], by reviewing 24 bronchoscopists, ranging from beginner to experienced, and discovered that the EBUS-STAT could be used to reliably score and classify clinicians from novice to expert [11-13].

EBUS-TBNA is proven to be safe and efficient and has replaced the prior gold standard, mediastinoscopy, for the diagnosis and staging of lung cancer and other causes of mediastinal and hilar adenopathy. Our study found that a single day (three hours) of EBUS-TBNA training using the EBUS-STAT exam and pre- and post-EBUS-SAT questionnaire with high-fidelity simulator and cases increased the confidence of both the first- and third-year pulmonary fellows in performing EBUS-TBNA procedures (p = 0.002 and 0.0001, respectively). Overall, there was no significant difference in EBUS-STAT score according to year of training (p = 0.71).

This is particularly interesting because the third-year fellows had more basic bronchoscopy experience and had completed many EBUS-TBNA procedures prior to the training session and indicates that our training approach may be beneficial for learners at multiple levels. The training session was well received by all fellows and required minimal planning and resources.

Current guidelines advocate a shift from mandating a specific minimum number of EBUS-TBNA procedures. Instead, they endorse an approach centered on skill-based assessments, such as the EBUS-STAT, along with comprehensive high-fidelity training [14,17]. Following such training, learners should continue to receive instruction and evaluation by expert clinicians on multiple patient procedures, to assess their proficiency in EBUS-TBNA, as the number of procedures required to achieve competence will vary.

This study has limitations. It was conducted at a single tertiary care academic center by a single instructor, and a limited number of pulmonary fellow trainees were included for analysis in both the first- and third-year groups (total N = 18). Further studies with a broader scope could be considered. In comparing the results of the EBUS-STAT exam between the first- and third-year pulmonary fellows, the number in each group was unbalanced (first year, N = 10 vs third year N = 8); thus, the conclusions for our secondary objectives were based on limited numbers of trainees.

## Conclusions

Our overall experience using the EBUS-SAT questionnaire and EBUS-STAT 10-item exam with our EBUS-TBNA course has been excellent, well-received by trainees, and will continue to be implemented for our pulmonary fellows in training at the end of the first year. Our teaching method enhances the understanding of trainees about the rationale behind EBUS-TBNA, as well as the procedural and technical skills required for it. As simulation training alone is insufficient to achieve competency, this approach is followed by continued training on patients and evaluation by expert clinicians during the second and third years of pulmonary fellowships. This training format may shorten the learning curve and number of procedures required to become competent, thus improving patient outcomes. Overall, recommendations are to move away from the traditional volume approach alone for EBUS-TBNA training and toward a more formal, structured teaching method using a skills-based assessment exam, such as the EBUS-STAT, along with simulation training, followed by training and evaluation by a skilled operator on patients. This method is supported and has been validated in multiple publications and should be incorporated into fellowship training.
